# Characterization of Lung Inflammatory Response to *Aspergillus fumigatus* Spores

**DOI:** 10.3390/jof9060682

**Published:** 2023-06-17

**Authors:** Alexandra Bouyssi, Tanguy Déméautis, Alexis Trecourt, Marie Delles, Fany Agostini, Guillaume Monneret, Olivier Glehen, Martine Wallon, Florence Persat, Gilles Devouassoux, Abderrazzak Bentaher, Jean Menotti

**Affiliations:** 1 UR3738 Centre pour l’lnnovation en Cancérologie de Lyon, Team Inflammation and Immunity of the Respiratory Epithelium, Claude Bernard University—Lyon 1, 69495 Pierre Bénite, France; alexandra.bouyssi@univ-lyon1.fr (A.B.);; 2 Department of Pathology, South Lyon Hospital, Hospices Civils de Lyon, 69495 Pierre Bénite, France; 3 Immunology Laboratory, EA7426, Edouard Herriot Hospital, Hospices Civils de Lyon and Claude Bernard University—Lyon 1, 69003 Lyon, France; 4 UR3738 Centre pour l’lnnovation en Cancérologie de Lyon, Surgical Department, South Lyon Hospital, Hospices Civils de Lyon, Claude Bernard University—Lyon 1, 69495 Pierre Bénite, France; 5 Department of Medical Mycology and Parasitology, Institute of Infectious Agents, Croix-Rousse Hospital, Hospices Civils de Lyon, 69004 Lyon, France; 6 Department of Pulmonology, Croix-Rousse Hospital, Hospices Civils de Lyon, 69004 Lyon, France

**Keywords:** *Aspergillus fumigatus*, spores, lung, macrophages, epithelial cell, immune response

## Abstract

The airway exposure to *Aspergillus fumigatus* spores (AFsp) is associated with an inflammatory response, potentially leading to allergic and/or chronic pulmonary aspergillosis. The aim of our study is to better understand the host response, first in vitro, then in vivo, following the chronic exposure of mice to AFsp. We investigated the inflammatory response to AFsp in cell mono- and co-culture systems with murine macrophages and alveolar epithelial cells. The mice were subjected to two intranasal instillations using 10^5^ AFsp. Their lungs were processed for inflammatory and histopathological analyses. In cell culture, the gene expressions significantly increased for TNF-α, CXCL-1, CXCL-2, IL-1β, IL-1α and GM-CSF in macrophages, with these increases being limited for TNF-α, CXCL-1 and IL-1α in epithelial cells. In co-culture, increases in the TNF-α, CXCL-2 and CXCL-1 gene expressions were observed to be associated with increased protein levels. The in vivo lung histological analyses of mice challenged by AFsp showed cellular infiltrates in the peribronchial and/or alveolar spaces. A Bio-Plex approach on the bronchoalveolar lavage revealed significant increases in the protein secretion of selected mediators of the challenged mice compared to the unchallenged mice. In conclusion, the exposure to AFsp resulted in a marked inflammatory response of macrophages and epithelial cells. These inflammatory findings were confirmed in mouse models associated with lung histologic changes.

## 1. Introduction

*Aspergillus fumigatus* is a filamentous airborne saprophytic and ubiquitous fungus. The exposure to its spores was associated with lung airway inflammation in subjects living in damp dwellings or exposed to professional/industrial environments [1,2], leading to the development of severe complications such as allergic bronchopulmonary aspergillosis (ABPA) or chronic pulmonary aspergillosis (CPA) [3,4]. Patients with chronic lung diseases such as chronic obstructive pulmonary disease (COPD), cystic fibrosis (CF) or chronic asthma are particularly exposed to *Aspergillus* complications, which worsens their symptoms and contributes to increased morbidity and mortality [5,6]. The worldwide incidence of ABPA is estimated to be around 5 million of 193 million adults with asthma, and among them, it is estimated that 400,000 also have CPA [7]. Chronic aspergillosis may evolve, at the final state, into invasive pulmonary aspergillosis (IPA), the disease observed in severely immunocompromised patients that are exposed to *A. fumigatus* spores such as neutropenic patients [8]. Of relevance as well, critically ill patients with severe flu or COVID-19 are at high risk of developing an associated pulmonary aspergillosis [9,10]. Furthermore, the increased use of immunosuppressants such as corticosteroids, anticancer chemotherapies and biotherapies in recent decades resulted in a higher worldwide incidence of aspergillosis [11,12].

In some patients, especially those with asthma, a link between a sensitization to fungi and the severity of the disease and frequency of exacerbations was described, contributing to poor clinical outcomes. In immunocompetent patients, daily inhaled *Aspergillus fumigatus* spores are usually eliminated via mucociliary clearance and resident macrophages, which are actors of the innate immune mechanisms [5,13,14,15]. Resident macrophages recognize different fungal molecular motifs such as galactomannan, chitin and β-1,3-glucan through different molecular motif recognition receptors [16]. Both the lung inflammatory response to *Aspergillus fumigatus* and the underlying mechanisms of ABPA or CPA development in the immunocompetent host are not completely understood.

Thus, the aim of the present study is to improve our understanding of the lung response to *A. fumigatus* spores. To this end, we first set up models of cell mono- and co-cultures using relevant lung cells, namely, alveolar epithelial cells and macrophages, that were exposed to *A. fumigatus* spores (AFsp), and we characterized their inflammatory responses to AFsp. Then, we developed a mouse model of the sensitization to AFsp, with week 1 and week 6 intranasal instillations of the spores, to further investigate the changes in the host lung inflammation and tissue.

Our study highlighted a strong inflammatory response in the monocultures of RAW 264.7 macrophages and the MLE-15 epithelial cells, and in the co-cultures of both cells. Interestingly, the cell response was different for the cells that were in monocultures compared with the cells that were in co-cultures, with a reduction in the expression of IL-1α in the co-culture compared to RAW 264.7 alone, and in contrary, a synergistic effect was observed for TNF-α, which was more increased in the co-cultured cells versus the monocultured cells. For the in vivo model, the mice elicited a robust inflammatory response, which was correlated with the pathological changes in the lung tissue.

## 2. Materials and Methods

Cell monocultures: Murine RAW 264.7 macrophages (ATCC^®^ TIB-71™, CLS Cat# 400319/p462_RAW-2647, RRID:CVCL_0493) and MLE-15 lung epithelial cells (kindly provided by Jeffrey Wittset and Lhousseine Touqui, RRID:CVCL_D581) were cultured in DMEM culture media (Life Technologies, Carlsbad, CA, USA) supplemented with 10% fetal bovine serum (Eurobio Scientific, Les Ulis, France) and 1% Penicillin/Streptomycin (Life Technologies) at 37 °C in a humidified atmosphere containing 5% CO_2_. Cells were washed with sterile phosphate-buffered saline (PBS) (Life Technologies), then treated at sub-confluence (80%) with *A. fumigatus* spores at a 1:3 ratio (cell/spores). *A. fumigatus* CBS144.89 strain spores were prepared as previously described [17]. Briefly, spores were grown from frozen stocks on Sabouraud + Chloramphénicol agar tubes (BioMérieux, Marcy l’Etoile, France) for 7 days at 37 °C. Resting conidia were then harvested with PBS containing 001% Tween 20 (Sigma-Aldrich^®^, St. Louis, MO, USA). The suspension was counted by using a Kova^®^ hemacytometer and calibrated to achieve the 1:3 ratio (cells:spores). A *Pseudomonas aeruginosa* lipopolysaccharide (LPS) at 1 μg/mL served as a positive control. Four hours post-treatment, RNA extraction and RT-qPCR were performed on cells. For each condition, cells were stained with Kwik-Diff Stain kit for cytologic analysis (Thermo Fisher, Waltham, MA, USA). Three independent experiments for both monocultures were performed.

Cell co-culture model: Two days before treatment, epithelial cells were seeded at 3 million per well in 12-well plates and incubated overnight. One day before treatment, macrophages were added to sub-confluent epithelial cells at a 1:2 ratio (macrophages: epithelial cells), after epithelial cell count. On treatment day, the cells were subjected to similar treatment with *A. fumigatus* as above. Four independent experiments were performed.

In parallel experiments, for co-culture control, cells were fixed with methanol and immunostained using the specific antibodies anti-CD68 (ABCAM, Cambridge, UK) for the macrophages and anti-E-cadherin (R&D Systems, Minneapolis, MN, USA) for epithelial cells. For secondary antibodies, Alexa Fluor™ 594 and 488 (Thermo Fisher) were used, respectively. Four independent experiments were performed.

Animal experiments: A total of 14 C57BL/6J Specific and Opportunistic Pathogen Free (SOPF) mice (8 males and 6 females, 14 weeks old, MGI Cat# 2159769, RRID: MGI: 2159769) were purchased from Charles River Laboratories. Mice were housed in ventilated racks for animal facilities with food and water ad libitum and a 12 h dark/light cycle. After a week of acclimatization period, mice were randomly divided into the following two groups with equal sex ratio: a control group exposed to two intranasal instillations of phosphate-buffered saline (PBS) (control group), and a spore group exposed to two intranasal instillations of *Aspergillus fumigatus* spores (AFsp group), both at week 1 and week 6. Mice were sacrificed 24 h after the last instillation, and their blood (serum), bronchoalveolar lavage (BAL) and lungs were recovered as previously described to perform inflammatory (left lobe) and histopathological analyses (right lobes) [18].

Ethical considerations: All animal experiments complied with the European Union Directive 2010/63/EU on the protection of animals used for scientific purposes. The protocol was approved by the French Ministry of Higher Education and Research after agreement from the local Ethical Committee for experiments on animals (approval number CECCAPP_LS_2017_016).

*Aspergillus fumigatus* spore preparation and intranasal instillation: *A. fumigatus* CBS144.89 strain spores were prepared as described for cell culture experiments [17]. The suspension was counted using a Kova^®^ hemacytometer and calibrated to a final concentration of 10^5^ conidia/50 µL in PBS + 0.01% Tween 20. The spore suspension obtained was administered via intranasal instillation in the two nares of the mice, after mice were anesthetized with isoflurane at 3%.

*Aspergillus* DNA detection: DNA extraction from lung was performed using an automaton Maxwell^®^16 (Promega, Madison, WI, USA) after grinding by using Roche MagNA Lyser (Roche, Bâle, Swiss) beads and eluted in 100 µL. DNA extracts were tested in duplicate with an *A. fumigatus*-specific qPCR as previously reported in [19] by using previously reported primers and probe [20]. For quantification, five serial ten-fold dilutions of *A. fumigatus* spores were used, and a standard curve was established between the threshold cycle (Ct) values and the spore numbers; this standard curve was used to estimate the spore number via interpolation of the Ct value obtained using real-time PCR [19].

Histopathological analysis: Pulmonary samples (right lobes) were processed for all 14 mice included. All samples were fixed in formalin (buffered formaldehyde 4%, pH 7, Laurypath, Brignais, France), split in four sections and embedded in paraffin (Tissue-TEK Paraffin Wax TEKK III, Sakura, Osaka, Japan). For each sample, two 3 µm thick tissue sections of the paraffin block were performed. A histopathological analysis was performed on both tissue sections by a pathologist (A.T) and an experienced researcher (A.Be.), blind to the randomization of mice, after staining with Hematoxylin–Eosin–Saffron (HES) stain (Tissue-Tek Prisma & Glas G2, Sakura) for tissue analysis, and Grocott stain for fungal morphology analysis (Slide stainer, Ventana Medical System, Tucson, Arizona, AZ, USA) according to the manufacturer’s recommendations. The microscopic analysis included the description of the following: presence/absence of tissue inflammation, location of the inflammation, type of inflammatory infiltrate (lymphocytes, plasma cells, macrophages, neutrophils, eosinophils) and semi quantitative inflammation quantification (mild: inflammatory cells easy to count, moderate: inflammatory cells difficult to count, severe: inflammatory cells impossible to count), presence/absence of perivascular edema and presence/absence of fungal elements on the slide stained via Grocott. Two independent readers read all slides.

All histopathological photographs for original figures of the present article were taken after slide scanning using Aperio AT2 scanner (Leica Biosystems, Nussloch, Germany) and Image Scope software (Leica Biosystems).

Gene expression quantification of inflammatory cytokines: RNA extraction from in vivo lung tissue or cultured cells was performed using TRIzol™ Reagent (Sigma-Aldrich^®^) and Nucleospin RNA set for NucleoZOL (Macherey-Nagel, Hoerdt, France) according to the manufacturer’s protocol. DNase free kit (Thermo Fisher) was used to eliminate residual gDNA. cDNA was obtained via reverse transcription using High-Capacity cDNA Reverse transcription kit (Thermo Fisher). TaqMan^®^ real-time quantitative polymerase chain reaction (RTqPCR) assays were performed using the corresponding cDNAs on an AriaMx Real-Time PCR thermocycler (Agilent Technologies, Vénissieux, France). The hypoxanthine-guanine phosphoribosyltransferase (HPRT) gene was used as endogenous control (Table S1). Results were expressed using the 2^−ΔΔCt^ method [21]. Of note, the Ct was set to 40 for *CXCL-1* and *GM-CSF* genes that were not expressed in untreated cells.

Enzyme-linked immunosorbent assay (ELISA): ELISA MAX™ Deluxe Set on TNF-α, IL-1α and IgE (BioLegend^®^, San Diego, CA, USA), and Quantikine^®^ CXCL-1 and CXCL-2 (BioTechne^®^, Minneapolis, MN, USA) were performed using supernatants collected from co-culture cell experiments or BAL samples (CXCL-2 and IgE) from mice according to the manufacturer’s protocol.

Multiplex immunoassays: Mouse BAL and sera were subjected to a multiplex immunoassay using Bio-Plex™ Pro Mouse Cytokine 23-plex Assay (Biorad™, Marnes-la-Coquette, France). The assays were performed by following the manufacturer’s protocol. The BAL were not diluted, and sera were diluted to a quarter.

Cytotoxicity: Cytotoxicity assay was performed using CyQUANT™ LDH Cytotoxicity Assay kit (Thermo Fisher). Assays were performed on supernatants of co-cultured cells and on BAL from spore and control groups following the manufacturer’s protocol.

Statistics: All statistical analyses were performed using Prism version 8.4.2 software (GraphPad Software, La Jolla, CA, USA), and data were represented as mean with standard deviation (SD) of minimum three independent experiments.

Statistical analyses were made on ΔCt for RT-qPCR and concentrations (pg/mL) for ELISA and BioPlex assays. To compare the difference between the 2 groups, normality was verified using Shapiro–Wilk test; then, either Student’s unpaired t-test or Welch’s test or Mann–Whitney test were performed. A *p* < 0.05 was considered statistically significant.

## 3. Results

### 3.1. Transcript Levels of Different Inflammatory Mediators in AFsp-Treated Monocultures

To assess the inflammatory response of the first sentinel cells involved in the immune reaction, we evaluated the mRNA expression profile of the different cytokines in the AFsp-exposed RAW 264.7 macrophages and the MLE-15 epithelial cells. The LPS-treated cells were used as positive controls (Supplementary Figure S3A,B).

**Figure 1:**
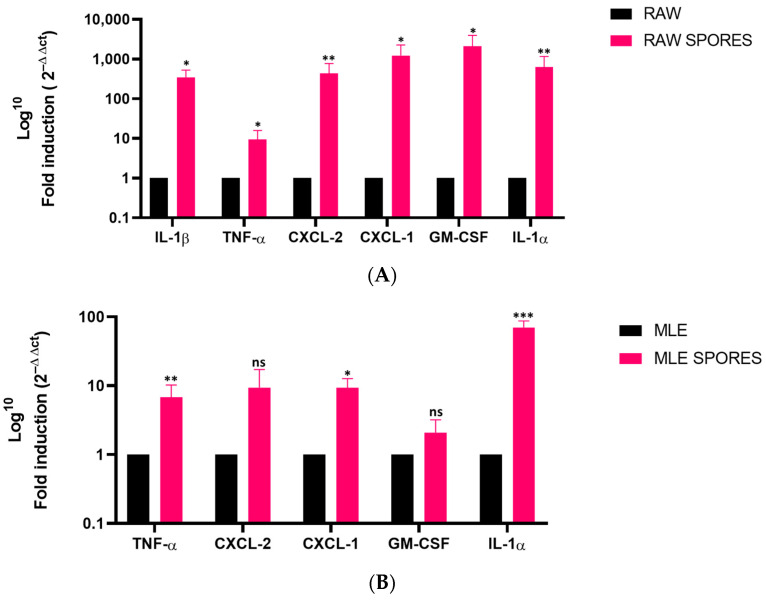
Gene expression of defined inflammatory mediators after a 4 h exposure of RAW 264.7 and MLE-15 cells to *A. fumigatus* spores. (**A**) Quantification of the gene expression of inflammatory markers in RAW 264.7 cells via RT-qPCR. (**B**) Quantification of the gene expression of inflammatory markers in MLE-15 cells via RT-qPCR. (*** *p* < 0.001; ** *p* < 0.01; * *p* < 0.05 versus untreated cells, *n* = 3; ns, not significant.) Data are represented as mean with SD.

In the RAW 264.7 cells, significant gene expression increases were observed for IL-1β (350-fold, *p* = 0.0179), TNF-α (9-fold, *p* = 0.0215), CXCL-2 (432-fold, *p* = 0.003), CXCL-1 (1200-fold, *p* = 0.0392), GM-CSF (2100-fold, *p* = 0.0196) and IL-1α (625-fold, *p* = 0.0035) after the exposure to *A. fumigatus* spores (Figure 1A).

In the MLE-15 cells, significant gene expression increases were observed for TNF-α (6-fold, *p* = 0.0065), CXCL-1 (9-fold, *p* = 0.0353) and IL-1α (70-fold, *p* = 0.0003) after the exposure to *A. fumigatus* spores (Figure 1B). No IL-1β expression could be detected in the untreated nor the treated MLE-15 cells. Although not statistically significant, an increasing trend in the CXCL-2 gene expression (9-fold, *p* = 0.0898) was observed.

### 3.2. Induced Gene Expression of Cytokines in RAW 264.7 and MLE-15 Cell Co-Culture

To explore the immune response to AFsp in more physiologic conditions to closely mimic the cross-talk between the two cell types, we set up a co-culture model of both the RAW 264.7 macrophages and the MLE-15 epithelial cells.

**Figure 2:**
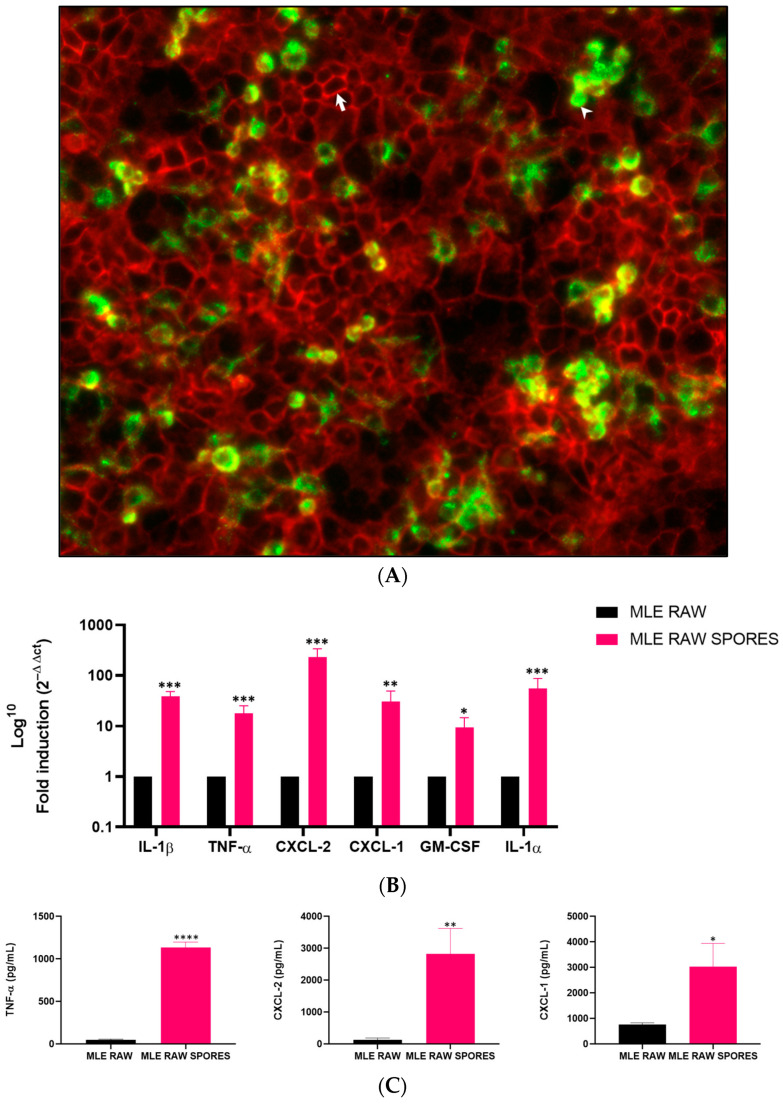
Immunofluorescence micrograph (×250 magnification) of the co-culture system (**A**); the white arrow points to E-cadherin staining for epithelial cells in red, and the arrowhead depicts CD-68 staining for macrophages in green-yellow (ratio: 2 MLE-15/1 RAW264.7). Gene expression of inflammatory mediators (**B**) and cytokine secretion in the supernatants after a 4 h exposure of the co-culture model to *A. fumigatus* spores (**C**) (**** *p* < 0.0001, *** *p* < 0.001, ** *p* < 0.01, * *p* < 0.05 versus untreated cells, *n* = 4). Data are represented as mean with SD.

The co-culture system was validated via fluorescence detection of both cell types, with the RAW 264.7 cells in green-yellow fluorescence and the MLE-15 cells in red (Figure 2A). The LPS-treated cells were used as positive controls (Supplementary Figure S3C). Interestingly, in response to *A. fumigatus*, significant increases in the gene expression of the defined mediators were observed with inductions of 38-fold for *IL-1β* (*p* = 0.0003), 17-fold for *TNF-α* (*p* = 0.0005), 233-fold for *CXCL-2* (*p* = 0.0003), 31-fold for *CXCL-1* (*p* = 0.0039), 9-fold for *GM-CSF* (*p* = 0.0110) and 55-fold for *IL-1α* (*p* = 0.0004) (Figure 2B).

### 3.3. Involvement of TNF-α, CXCL-2 and CXCL-1 in Anti-Aspergillus Response in Co-Culture Model

To determine whether the protein levels corroborate the transcript expression of the genes of interest, we performed ELISA on selected mediators. The LPS-treated cells were used as positive controls (Supplementary Figure S3D). Significant protein secretion was detected for TNF-α (24-fold, *p* < 0.0001), CXCL-2 (22-fold, *p* = 0.0063) and CXCL-1 (4-fold, *p* = 0.0152) (Figure 2C). However, IL-1α showed no significant secretion compared to the untreated cells. A cytotoxicity assay was performed on the supernatant of the co-cultured cells and it showed no statistically significant difference (*p* = 0.7326) in the percentage of cellular viability between the controls and the cells exposed to the spores (Figure S1).

After the acute exposure of macrophages and epithelial cells to AFsp, we aimed to study the inflammatory response of sensitized mice to AFsp after two instillations 5 weeks apart, mimicking repeated exposures with a long follow-up of these immunocompetent mice that are not susceptible to *Aspergillus* invasive infection.

### 3.4. Effects on Mouse Body Weight

No statistical difference was observed in the mouse body weight between the control group and the mice exposed to two intranasal instillations of *Aspergillus fumigatus* spores. No statistical difference was observed in the sex-dependent comparison either (Figure S2A,B).

### 3.5. Tissue Inflammation and Perivascular Edema Induced via AFsp Exposure

**Figure 3:**
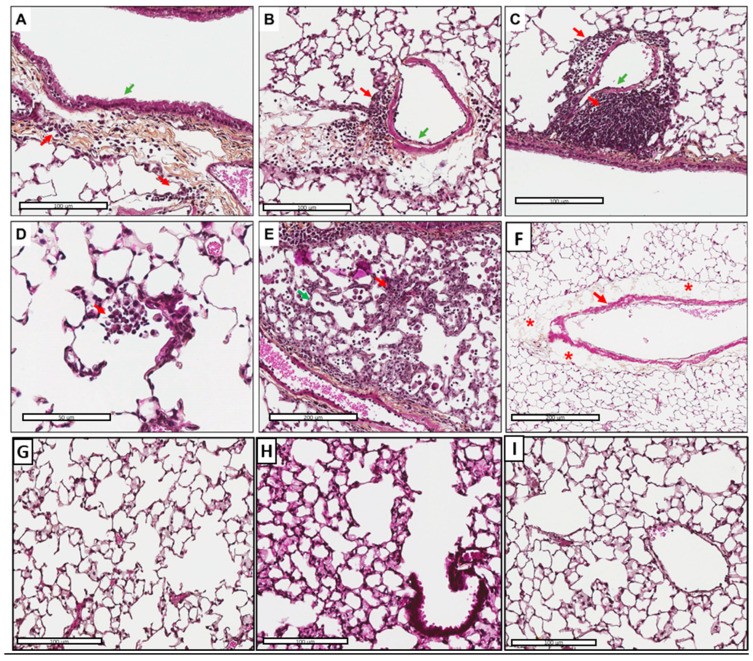
Histopathological features of inflammatory reaction (location and intensity) in mice challenged with *A. fumigatus* spores (**A**–**F**) and control mice (**G**–**I**). (**A**) (Hematoxylin–eosin–saffron (HES), ×200 magnification). Mild peribronchiolar inflammation (red arrows): inflammatory cells easy to count around the bronchioles (green arrow). (**B**) (HES, ×200 magnification). Moderate perivascular inflammation (red arrow): inflammatory cells difficult to count around the vascular wall (green arrow). (**C**) (HES, ×200 magnification). Severe perivascular inflammation (red arrow): inflammatory cells impossible to count around the vascular wall (green arrow). (**D**) (HES, ×400 magnification). Mild alveolar inflammation with lymphocytes, plasma cells and few neutrophils and eosinophils (red arrow). (**E**) (HES, ×190 magnification). Moderate alveolar inflammation (red arrow) with lymphocytes, plasma cells and macrophages with thickening of the alveolar walls (green arrow). (**F**) (HES, ×100 magnification). Perivascular edema: edema (red asterisks) surrounding the vessel wall (red arrow). (**G**–**I**) (HES, ×200 magnification). No alveolar or peribronchiolar inflammation (except in 1 mouse out of 7), no fibrosis and no edema were observed in the control group.

Although the mice had the same weight and showed no outward signs of stress, the exposure of the mice to AFsp led to inflammation in 6/7 (87.5%) cases (Figure 3A–E). The inflammatory reaction was composed of lymphocytes, plasma cells and some neutrophils/eosinophils with a peribronchiolar, alveolar and perivascular location in all cases (100%, 6/6). A perivascular edema was observed in 7/7 (100%) cases (Figure 3F). No fungal elements, hypercrinia or fibrosis were observed on the Grocott and on the HES slides.

In the control group, inflammation was observed in only 1/7 (14.3%) cases. The inflammatory reaction was composed of lymphocytes and plasma cells and was perivascular and peribronchiolar. No perivascular edema were observed (Figure 3G–I). All the results are summarized in Table S2.

### 3.6. Inflammatory Cell Recruitment, Especially Macrophages in Response to AFsp

**Figure 4:**
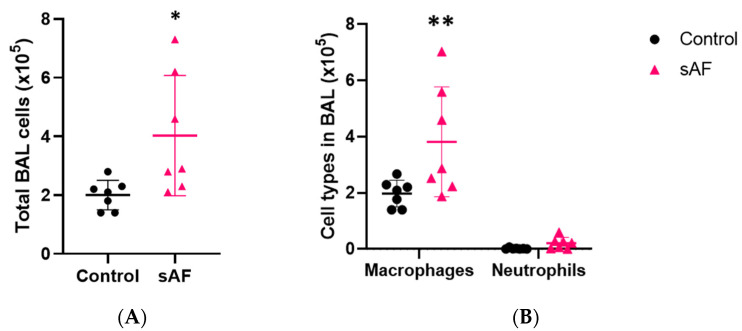
Total BAL cell counts (**A**) and cell types in BAL (**B**) from control mice or mice exposed to spores (** *p* < 0.01; * *p* < 0.05). Total cell counts in the BAL was measured using an Automatic Cell Counter with trypan blue. The cellularity was estimated on cytospin slides. Data are represented as mean with SD.

To explore the underlying basis of the inflammatory reaction in tissue, we investigated the inflammatory cell recruitment in the mice exposed to AFsp. The total cell counts in the BAL (Figure 4A) of the mice exposed to the spores were significantly increased (*p* = 0.0399) compared to the control group. Macrophages represented the dominant cell type and was significantly increased in the AFsp group compared to the control group (Figure 4B, *p* = 0.0046).

### 3.7. Quantification of Lung Fungal Burden

**Figure 5:**
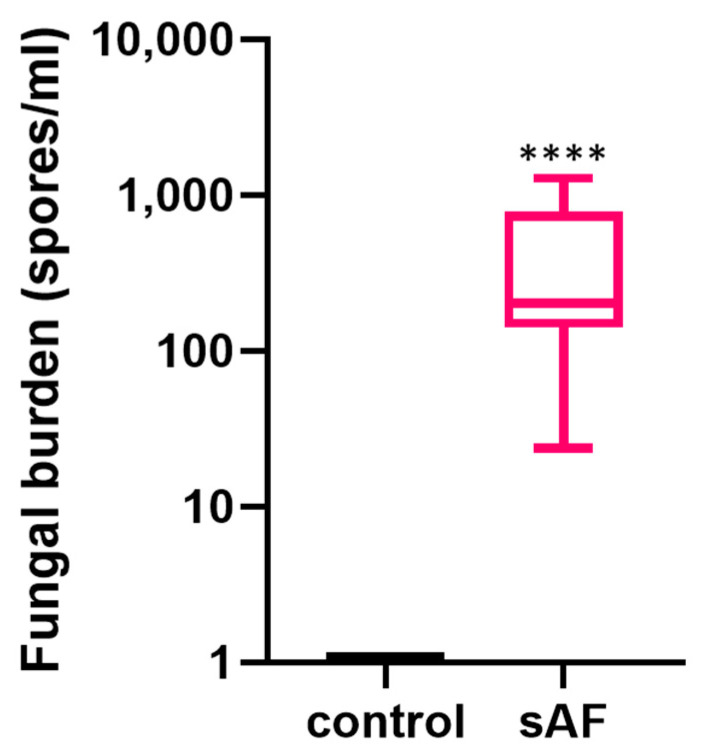
Fungal burden in mouse lung after exposure to two instillations of 10^5^ *A. fumigatus* spores (sAF) 5 weeks apart (**** *p* < 0.0001). Data are represented as Min to Max in box and whiskers form.

The presence of *A. fumigatus* spores was evidenced by *A. fumigatus*-specific qPCR in the lungs of the mice exposed to the spores, whereas no fungal DNA was observed in the control group. The mean fungal burden in the mice challenged with *A. fumigatus* spores was 428.8 spore-equivalents/mL (Figure 5).

### 3.8. Increased Transcript Levels of Some Inflammatory Mediators in AFsp-Exposed Lungs

**Figure 6:**
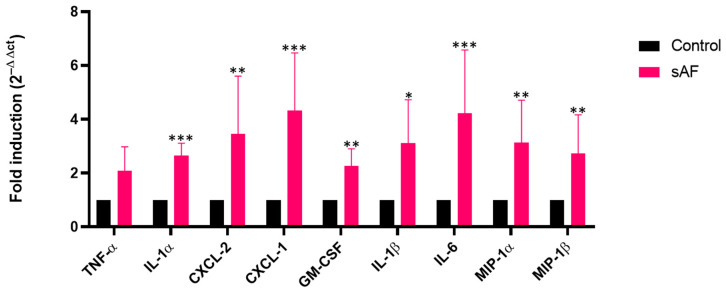
Gene expression of defined inflammatory mediators after exposure of mice to two instillations of 10^5^ *A. fumigatus* spores (sAF) 5 weeks apart (*** *p* < 0.001, ** *p* < 0.01, * *p* < 0.05 versus control mice, *n* = 7). Data are represented as mean with SD.

To corroborate the results obtained in vitro, we tested the levels of gene expression of the same mediators in the lungs. In the mice exposed to *A. fumigatus* spores compared to the control mice, significant gene expression increases were evidenced (Figure 6) for *CXCL-2* (3.5-fold, *p* = 0.0043), *IL-1α* (2.7-fold, *p* = 0.0007), *CXCL-1* (4-fold, *p* = 0.0003), *GM-CSF* (2-fold, *p* = 0.0050), *IL-1β* (3-fold, *p* = 0.0379), *IL-6* (4-fold, *p* = 0.0005), *MIP-1α* (3-fold, *p* = 0.0054), and *MIP-1β* (2.7-fold, *p* = 0.0022). Although not statistically significant, an increasing trend in the *TNF-α* gene expression (2-fold, *p* = 0.0829) was observed.

### 3.9. Pattern of Several Inflammatory Mediators in BAL from Challenged Mice

To further explore the inflammatory response, we assessed the levels of secretion of several mediators in the BAL of the AFsp mice using a multiplex immunoassay approach.

**Figure 7:**
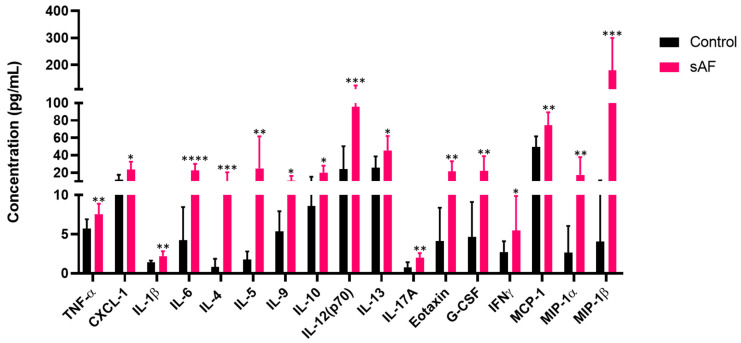
Quantification of defined secreted cytokines in bronchoalveolar lavage (BAL) of mice exposed to *A. fumigatus* spores (sAF) (**** *p* < 0.0001, *** *p* < 0.001, ** *p* < 0.01, * *p* < 0.05 versus control mice, *n* = 7). Data are represented as mean with SD.

Significant increases in the secretion of 17 proteins were found in the BAL (Figure 7) from the mice challenged with the spores. The results are summarized in Table S3, with the higher increase (36-fold) being observed for MIP-1β. A cytotoxicity test with LDH release in the BAL from the mice showed no statistically significant difference (*p* = 0.3) and ELISA showed an absence of IgE in the BAL (unpublished work, Bouyssi et al., Claude Bernard University, Lyon, France; materials as described in the materials and methods section, 2023).

### 3.10. Increased Protein Levels of Inflammatory Mediators in Sera from Challenged Mice

**Figure 8:**
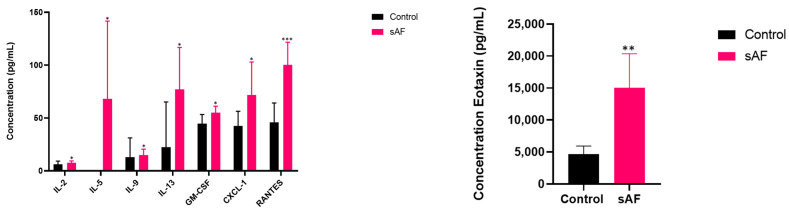
Quantification of defined secreted cytokines in sera of mice exposed to *A. fumigatus* spores (sAF) (*** *p* < 0.001, ** *p* < 0.01, * *p* < 0.05 versus control mice, *n* = 7). Data are represented as mean with SD.

To assess the systemic inflammation, we tested the sera from the AFsp and control mice groups using the same multiplex immunoassay. A significant increase in the secretion of several proteins was observed in the AFsp mice (Figure 8) as follows: IL-2 (1.2-fold, *p* = 0.0490), IL-5 (68-fold, *p* = 0.0496), IL-9 (1.13-fold, *p* = 0.0233), IL-13 (3.4-fold, *p* = 0.0338), GM-CSF (1.2-fold, *p* = 0.0224), CXCL-1 (1.7-fold, *p* = 0.0410), RANTES (=CCL5) (2.2-fold, *p* = 0.0003) and Eotaxin (3.3-fold, *p* = 0.0017).

## 4. Discussion

To better understand the pulmonary and systemic inflammatory response of immunocompetent hosts to *A. fumigatus* spores, we developed the following models: monocultures then co-cultures with macrophages and epithelial cells, relevant lung cells involved in immune response and an animal model. The AFsp exposure was performed in the culture model while the sensitization to AFsp was mimicked in the mouse model with two AFsp instillations 5 weeks apart, with a long mouse follow-up (6 weeks).

We assessed the inflammatory response of the murine macrophages and alveolar epithelial cells, individually or co-cultured, to AFsp. Our rationale was to closely mimic the cross-talk, if any, between these two cell types that are known to act as sentinels for the immune system in the lungs.

Our results highlighted, as expected, a strong induction of proinflammatory cytokine gene expression (*IL-1α*, *IL-1β*, *CXCL-2*, *TNF-α*, *CXCL-1* and *GM-CSF*) via murine macrophages, which are key cells for the innate immune response. A weaker induction of proinflammatory cytokine gene expression (*IL-1α*, *CXCL-1* and *TNF-α*) was observed for the epithelial cells, compared to the macrophages, following exposure to AFsp. All of these mediators are involved in immune cell recruitments, especially neutrophils [22,23,24,25], which are essential cells for the clearance of *A. fumigatus* from the airway [26]. In the study conducted by Pylkkänen et al., the exposure of mouse macrophages to 10^7^ environmental *A. fumigatus* spores led to a significant increase in the TNF-α gene expression, with the maximal at 6 h with a two-fold induction, and no change in the IL-1β gene expression regardless of the duration of treatment and the concentrations of spores [27]. In our study, a 4 h exposure was sufficient to obtain a 9-fold induction for the TNF-α gene expression and a 350-fold induction for the IL-1β gene expression with the same cells (RAW 264.7 macrophages). In the article by Bellanger et al. on the A549 human lung epithelial cell line, a significant induction of the IL-8 (homologous to CXCL-1 and CXCL-2 in mice), TNF-α and GM-CSF gene expressions was found at 8 h and 24 h, but no significant induction was observed before 8 h [28]. In our study, with the murine epithelial cells, after a 4 h spore exposure, we were able to detect an increase in the TNF-α and CXCL-1 gene expressions. The treatment was stopped after 4 h because living conidia began to grow, and at 24 h, hyphae were present in the medium. TNF-α and CXCL-2 were secreted by the macrophages in response to the live spores after 18 h of treatment in the study conducted by Hohl et al. [29]. In our study, the macrophages elicited a strong response with a 430-fold induction for the CXCL-2 gene expression after 4 h. Moreover, our results and those of others [27,28,29] showed that epithelial cells and macrophages are key cells in response to *A. fumigatus* spores, and only 4 h was sufficient to start up an immune response in each monoculture. Additionally, we wanted to investigate the inflammatory response of the cells in the co-culture.

Our study evidenced a marked inflammatory response to the *A. fumigatus* spores in the co-culture model, with inductions of the *CXCL-2*, *IL-1α*, *IL-1β*, *CXCL-1*, *TNF-α* and *GM-CSF* gene expressions. Interestingly, the cell response to the *A. fumigatus* spores was different with the cells that were in the monocultures compared to those that were in the co-culture. Indeed, a reduction in the pattern of expression was evidenced in the co-culture model compared to the RAW 264.7 cells alone for cytokines such as IL-1α, which showed an 11-fold reduction in the gene expression induction in the co-culture when compared with the RAW 264.7 cells alone, and a 1.3-fold reduction when compared with the MLE-15 cells alone. However, cells can also have synergistic effects as with the TNF-α gene expression, which was 9-fold and 6-fold induced by the RAW 264.7 cells alone and by the MLE-15 cells alone, respectively, whereas it was 17-fold induced in the co-culture. This might be beneficial by yielding strong inflammatory and immune responses to eliminate a pathogen while reducing the deleterious effects of the cytokine storm. Moreover, IL-1α was not detected in the supernatants of the co-culture exposed to the spores, but LPS induced IL-1α secretion. In our study, the IL-1α gene expression was up-regulated, but indeed, post-transcriptional modifications may take place. It was highly up-regulated in the mice as well but was not found at the protein level (BioPlex) in the broncho-alveolar lavage either. It might not be secreted in this condition, or it might serve for other genes or the regulation of other proteins.

Most studies on *Aspergillus fumigatus* focused on one cell type in particular, either epithelial cells, macrophages or fibroblasts, but rarely on a more complex model, where several cell types are in contact to try to recreate the pulmonary environment. Our co-culture model makes it possible to study the inflammatory response after the exposure to fungal spores by approaching the in vivo model with the two cell types involved in the first line of defense against pathogens.

To better understand the pulmonary inflammatory response to *A. fumigatus* spores in immunocompetent hosts, mice were challenged with two exposures of 10^5^ spores 5 weeks apart. Most studies with the exposure of mice to *A. fumigatus* spores focused on an invasive pulmonary aspergillosis model in immunocompromised mice [30], or on an allergy model with the treatment with ovalbumin before or after AFsp challenge or allergen of *A. fumigatus* before using spores [31], or with purified recombinant *A. fumigatus* allergens [32,33], or used the intratracheal [34] or intraperitoneal inoculation of spores [35]. To our knowledge, there are no other studies that aim to assess the inflammatory response in immunocompetent mice challenged twice 5 weeks apart with resting conidia. This long-term in vivo model mimics chronic aspergillosis with the first contamination with sensitization, then spore challenge-inducing exacerbation. In this model, the mice elicited a strong inflammatory response, which was correlated with pathological changes in the lung.

Indeed, in histological analyses, inflammation was reported for 6/7 cases and composed of lymphocytes, plasma cells and some neutrophils/eosinophils, and perivascular edema was present for all of the mice exposed to the spores.

The inflammation was also supported in the lung by the expression of several genes such as *IL-1β*, *CXCL-2*, *CXCL-1*, *GM-CSF*, *IL-6*, *MIP-1α* and *MIP-1β*, and correlated with their up-secretion in the BAL.

Interestingly, in our in vivo study, the other mediators screened in the multiplex immunoassay were increased as follows: IL-4, a cytokine secreted particularly by eosinophils and which plays a role in the regulation of antibody production, is known to enhance the secretion and cell surface expression of IgE and IgG1 [36,37], and is critical to inflammation and pulmonary eosinophilia induced by *A. fumigatus* spores [38]; IL-5, a cytokine involved in the survival, differentiation and chemotaxis of eosinophils [39]; IL-13, a cytokine that plays an important role in allergic inflammation [40], stimulates B cell proliferation and activation of eosinophils, basophils and mast cells and is essential to pulmonary eosinophilia induced by *A. fumigatus* spores [38]; eotaxin, which promotes the accumulation of eosinophils in response to allergens, an important feature of allergic inflammation reactions [41]; MCP-1 (=CCL2), which has a chemoattractant activity on monocytes and basophils [42]; MIP-1α (=CCL3), which has a potent chemotactic activity on eosinophils [43]; MIP-1β (=CCL4), which is involved in eosinophil recruitment and is a biomarker of type 2 airway inflammation [44] and RANTES (=CCL5), a chemoattractant of blood monocytes, memory helper T-cells and eosinophils. RANTES also causes the release of histamine and activates eosinophils [45]. All of the mediators are particularly involved in eosinophil/basophil recruitment or are secreted by these cells. Eosinophils and basophils are important cells that are involved in allergic inflammation [46]. These mice appear to have a lot of signs that point to an allergic response after two exposures 5 weeks apart with significant inflammation and the presence of inflammatory cells such as lymphocytes, plasma cells, neutrophils and eosinophils. Indeed, such inflammation can lead to ABPA or CPA and exacerbate other inflammatory diseases such as chronic obstructive pulmonary disease or increase the risk of having a worsened lung function. Noverr et al. showed that, after antibiotic treatment, only two intranasal exposures to *A. fumigatus* spores 5 days apart are not sufficient to induce a pulmonary allergic response without a sensitizing event, but it was performed with a different strain of mice (BALB/c), with a different strain of spores (ATCC 13073) and with two-log more spores than in our experiment [47]. Mice can also be sensitized with fungal allergens before spore inhalation to create the allergic response [31]. In our study, two instillations 5 weeks apart appeared to be sufficient to yield an *A. fumigatus*-induced cytokine response with similarities to an allergic airway disease. However, most studies showed an increase in the IgE in the sera of mice exposed to *Aspergillus fumigatus*. In our study, we did not find these results in the BAL, but this might be due to the fact that the IgE half-life in adult mice is only 12 h [48]. It is important to understand the immune mechanisms involved in the response against *Aspergillus fumigatus* spores in immunocompetent hosts to develop new therapeutic solutions against the potentially deleterious immune response. The mechanism of inflammation is complex and involves both immune cells and epithelial cells, which are not only useful for mucociliary clearance, but also play a major role in inflammation.

## Data Availability

The data presented in this article are available in the article or in the Supplementary Material.

## Supplementary Materials

The following supporting information can be downloaded at https://www.mdpi.com/article/10.3390/jof9060682/s1, Table S1: TaqMan^®^ gene expression assays; Table S2: Histopathological findings; Table S3: Summary of inflammatory mediator increases in BAL of mice exposed to spores compared to controls with fold increases and *p*-values; Figure S1: Percentage of cellular viability on co-cultured cells; Figure S2: Weight of female (A) and male (B) mice after exposure to *Aspergillus fumigatus* spores; Figure S3: Gene expression and secretion of defined inflammatory mediators after a 4 h exposure of RAW 264.7, MLE-15 and co-cultured MLE-15 and RAW 264.7 cells to LPS at 1 μg/mL.

## References

[1] Smith N.L., Denning D.W. (2011). Underlying conditions in chronic pulmonary aspergillosis including simple aspergilloma. Eur. Respir. J..

[2] Lauruschkat C.D., Etter S., Schnack E., Ebel F., Schäuble S., Page L., Rümens D., Dragan M., Schlegel N., Panagiotou G. (2021). Chronic Occupational Mold Exposure Drives Expansion of Aspergillus-Reactive Type 1 and Type 2 T-Helper Cell Responses. J. Fungi.

[3] Camara B., Reymond E., Saint-Raymond C., Roth H., Brenier-Pinchart M.-P., Pinel C., Cadranel J., Ferretti G., Pelloux H., Pison C. (2015). Characteristics and outcomes of chronic pulmonary aspergillosis: A retrospective analysis of a tertiary hospital registry: Chronic pulmonary aspergillosis in non-immunocompromised patients. Clin. Respir. J..

[4] Denning D.W., Cadranel J., Beigelman-Aubry C., Ader F., Chakrabarti A., Blot S., Ullmann A.J., Dimopoulos G., Lange C. (2016). Chronic pulmonary aspergillosis: Rationale and clinical guidelines for diagnosis and management. Eur. Respir. J..

[5] Denning D.W. (2006). The link between fungi and severe asthma: A summary of the evidence. Eur. Respir. J..

[6] Bulpa P., Duplaquet F., Dimopoulos G., Vogelaers D., Blot S. (2020). Invasive Pulmonary Aspergillosis in Chronic Obstructive Pulmonary Disease Exacerbations. Semin. Respir. Crit. Care Med..

[7] Denning D.W., Pleuvry A., Cole D.C. (2013). Global burden of allergic bronchopulmonary aspergillosis with asthma and its complication chronic pulmonary aspergillosis in adults. Med. Mycol..

[8] Ledoux M.-P., Guffroy B., Nivoix Y., Simand C., Herbrecht R. (2020). Invasive Pulmonary Aspergillosis. Semin. Respir. Crit. Care Med..

[9] Dewi I.M., Janssen N.A., Rosati D., Bruno M., Netea M.G., Brüggemann R.J., Verweij P.E., van de Veerdonk F.L. (2021). Invasive pulmonary aspergillosis associated with viral pneumonitis. Curr. Opin. Microbiol..

[10] Dupont D., Menotti J., Turc J., Miossec C., Wallet F., Richard J.-C., Argaud L., Paulus S., Wallon M., Ader F. (2021). Pulmonary aspergillosis in critically ill patients with Coronavirus Disease 2019 (COVID-19). Med. Mycol..

[11] Bongomin F., Gago S., Oladele R., Denning D. (2017). Global and Multi-National Prevalence of Fungal Diseases—Estimate Precision. J. Fungi.

[12] Gangneux J.-P., Camus C., Philippe B. (2010). Epidemiology of invasive aspergillosis and risk factors in non neutropaenic patients. Rev. Mal. Respir..

[13] Black P.N., Udy A.A., Brodie S.M. (2000). Sensitivity to fungal allergens is a risk factor for life-threatening asthma: Fungal allergens and asthma. Allergy.

[14] Zureik M. (2002). Sensitisation to airborne moulds and severity of asthma: Cross sectional study from European Community respiratory health survey. BMJ.

[15] Goh K.J., Yii A., Lapperre T., Chan A., Chew F., Chotirmall S., Koh M. (2017). Sensitization to Aspergillus species is associated with frequent exacerbations in severe asthma. J. Asthma Allergy.

[16] Margalit A., Kavanagh K. (2015). The innate immune response to *Aspergillus fumigatus* at the alveolar surface. FEMS Microbiol. Rev..

[17] Persoz C., Leleu C., Achard S., Fasseu M., Menotti J., Meneceur P., Momas I., Derouin F., Seta N. (2011). Sequential air-liquid exposure of human respiratory cells to chemical and biological pollutants. Toxicol. Lett..

[18] Guyot N., Wartelle J., Malleret L., Todorov A.A., Devouassoux G., Pacheco Y., Jenne D.E., Belaaouaj A. (2014). Unopposed Cathepsin G, Neutrophil Elastase, and Proteinase 3 Cause Severe Lung Damage and Emphysema. Am. J. Pathol..

[19] Alanio A., Menotti J., Gits-Muselli M., Hamane S., Denis B., Rafoux E., Peffault de la Tour R., Touratier S., Bergeron A., Guigue N. (2017). Circulating Aspergillus fumigatus DNA Is Quantitatively Correlated to Galactomannan in Serum. Front. Microbiol..

[20] Challier S., Boyer S., Abachin E., Berche P. (2004). Development of a Serum-Based Taqman Real-Time PCR Assay for Diagnosis of Invasive Aspergillosis. J. Clin. Microbiol..

[21] Livak K.J., Schmittgen T.D. (2001). Analysis of Relative Gene Expression Data Using Real-Time Quantitative PCR and the 2^−ΔΔCT^ Method. Methods.

[22] Rider P., Carmi Y., Guttman O., Braiman A., Cohen I., Voronov E., White M.R., Dinarello C.A., Apte R.N. (2011). IL-1α and IL-1β Recruit Different Myeloid Cells and Promote Different Stages of Sterile Inflammation. J. Immunol..

[23] De Filippo K., Dudeck A., Hasenberg M., Nye E., van Rooijen N., Hartmann K., Gunzer M., Roers A., Hogg N. (2013). Mast cell and macrophage chemokines CXCL1/CXCL2 control the early stage of neutrophil recruitment during tissue inflammation. Blood.

[24] Jones M.R., Simms B.T., Lupa M.M., Kogan M.S., Mizgerd J.P. (2005). Lung NF-κB Activation and Neutrophil Recruitment Require IL-1 and TNF Receptor Signaling during Pneumococcal Pneumonia. J. Immunol..

[25] Laan M., Prause O., Miyamoto M., Sjöstrand M., Hytönen A.M., Kaneko T., Lötvall J., Lindén A. (2003). A role of GM-CSF in the accumulation of neutrophils in the airways caused by IL-17 and TNF-α. Eur. Respir. J..

[26] Cramer R.A., Rivera A., Hohl T.M. (2011). Immune responses against Aspergillus fumigatus: What have we learned?. Curr. Opin. Infect. Dis..

[27] Pylkkänen L., Gullstén H., Majuri M.-L., Andersson U., Vanhala E., Määttä J., Meklin T., Hirvonen M.-R., Alenius H., Savolainen K. (2004). Exposure to Aspergillus fumigatus spores induces chemokine expression in mouse macrophages. Toxicology.

[28] Bellanger A.-P., Millon L., Khoufache K., Rivollet D., Bièche I., Laurendeau I., Vidaud M., Botterel F., Bretagne S. (2009). Aspergillus fumigatus germ tube growth and not conidia ingestion induces expression of inflammatory mediator genes in the human lung epithelial cell line A549. J. Med. Microbiol..

[29] Hohl T.M., Van Epps H.L., Rivera A., Morgan L.A., Chen P.L., Feldmesser M., Pamer E.G. (2005). Aspergillus fumigatus Triggers Inflammatory Responses by Stage-Specific β-Glucan Display. PLoS Pathog..

[30] Leleu C., Menotti J., Meneceur P., Choukri F., Sulahian A., Garin Y.J.-F., Derouin F. (2013). Efficacy of liposomal amphotericin B for prophylaxis of acute or reactivation models of invasive pulmonary aspergillosis: Prophylaxis of invasive aspergillosis. Mycoses.

[31] Hoselton S.A., Samarasinghe A.E., Seydel J.M., Schuh J.M. (2010). An inhalation model of airway allergic response to inhalation of environmental *Aspergillus fumigatus* conidia in sensitized BALB/c mice. Med. Mycol..

[32] Kurup V.P., Xia J.-Q., Crameri R., Rickaby D.A., Choi H.Y., Flückiger S., Blaser K., Dawson C.A., Kelly K.J. (2001). Purified Recombinant *A. fumigatus* Allergens Induce Different Responses in Mice. Clin. Immunol..

[33] Mcmillan S.J., Lloyd C.M. (2004). Prolonged allergen challenge in mice leads to persistent airway remodelling. Clin. Htmlent Glyphamp Asciiamp Exp. Allergy.

[34] Hogaboam C.M., Blease K., Mehrad B., Steinhauser M.L., Standiford T.J., Kunkel S.L., Lukacs N.W. (2000). Chronic Airway Hyperreactivity, Goblet Cell Hyperplasia, and Peribronchial Fibrosis during Allergic Airway Disease Induced by Aspergillus fumigatus. Am. J. Pathol..

[35] Haczku A., Atochina E.N., Tomer Y., Chen H., Scanlon S.T., Russo S., Xu J., Panettieri R.A., Beers M.F. (2001). *Aspergillus fumigatus* -Induced Allergic Airway Inflammation Alters Surfactant Homeostasis and Lung Function in BALB/c Mice. Am. J. Respir. Cell Mol. Biol..

[36] Carr C., Aykent S., Kimack N.M., Levine A.D. (1991). Disulfide assignments in recombinant mouse and human interleukin 4. Biochemistry.

[37] Yokota T., Otsuka T., Mosmann T., Banchereau J., DeFrance T., Blanchard D., De Vries J.E., Lee F., Arai K. (1986). Isolation and characterization of a human interleukin cDNA clone, homologous to mouse B-cell stimulatory factor 1, that expresses B-cell- and T-cell-stimulating activities. Proc. Natl. Acad. Sci. USA.

[38] Dietschmann A., Schruefer S., Krappmann S., Voehringer D. (2020). Th2 cells promote eosinophil-independent pathology in a murine model of allergic bronchopulmonary aspergillosis. Eur. J. Immunol..

[39] Clutterbuck E., Hirst E., Sanderson C. (1989). Human interleukin-5 (IL-5) regulates the production of eosinophils in human bone marrow cultures: Comparison and interaction with IL-1, IL-3, IL-6, and GMCSF. Blood.

[40] Conde E., Bertrand R., Balbino B., Bonnefoy J., Stackowicz J., Caillot N., Colaone F., Hamdi S., Houmadi R., Loste A. (2021). Dual vaccination against IL-4 and IL-13 protects against chronic allergic asthma in mice. Nat. Commun..

[41] Gonzalo J.-A., Jia G.-Q., Aguirre V., Friend D., Coyle A.J., Jenkins N.A., Lin G.-S., Katz H., Lichtman A., Copeland N. (1996). Mouse Eotaxin Expression Parallels Eosinophil Accumulation during Lung Allergic Inflammation but It Is Not Restricted to a Th2-Type Response. Immunity.

[42] Weber M., Uguccioni M., Baggiolini M., Clark-Lewis I., Dahinden C.A. (1996). Deletion of the NH2-terminal residue converts monocyte chemotactic protein 1 from an activator of basophil mediator release to an eosinophil chemoattractant. J. Exp. Med..

[43] Rose C.E., Lannigan J.A., Kim P., Lee J.J., Fu S.M., Sung S.J. (2010). Murine lung eosinophil activation and chemokine production in allergic airway inflammation. Cell Mol. Immunol..

[44] Kobayashi Y., Konno Y., Kanda A., Yamada Y., Yasuba H., Sakata Y., Fukuchi M., Tomoda K., Iwai H., Ueki S. (2019). Critical role of CCL4 in eosinophil recruitment into the airway. Clin. Exp. Allergy.

[45] Alam R., Stafford S., Forsythe P., Harrison R., Faubion D., Lett-Brown M.A., Grant J.A. (1993). RANTES is a chemotactic and activating factor for human eosinophils. J. Immunol. Baltim. Md 1950.

[46] Stone K.D., Prussin C., Metcalfe D.D. (2010). IgE, mast cells, basophils, and eosinophils. J. Allergy Clin. Immunol..

[47] Noverr M.C., Falkowski N.R., McDonald R.A., McKenzie A.N., Huffnagle G.B. (2005). Development of Allergic Airway Disease in Mice following Antibiotic Therapy and Fungal Microbiota Increase: Role of Host Genetics, Antigen, and Interleukin-13. Infect. Immun..

[48] Vieira P., Rajewsky K. (1988). The half-lives of serum immunoglobulins in adult mice. Eur. J. Immunol..

