# Supplementary material for: Characterization of Lung Inflammatory Response to Aspergillus fumigatus Spores
Source: J Fungi (Basel). 2023 Jun 17;9(6):682. doi: 10.3390/jof9060682 (PMC10305343; doi:10.3390/jof9060682)
Supplement: Supplementary file 1 [file jof-09-00682-s001.zip › jof-2386950-supplementary.pdf]

SUPPLEMENTARY DATA:

Table S1. TaqMan® gene expression assays

Species	Gene	Assay ID	Provider
Mouse	*HPRT*	Mm03024075_m1	Life technologies
Mouse	*Il1β*	Mm00434228_m1	Life technologies
Mouse	*Il1α*	Mm00439620_m1	Life technologies
Mouse	*Cxcl1*	Mm04207460_m1	Life technologies
Mouse	*Cxcl2 (=MIP-2)*	Mm00436450_m1	Life technologies
Mouse	*Tnf*	Mm00443258_m1	Life technologies
Mouse	*Csf2 (=GM-CSF)*	Mm01290062_m1	Life technologies
Mouse	*Il6*	Mm00446190_m1	Life technologies
Mouse	*Ccl3 (= MIP-1α)*	Mm00441258_m1	Life technologies
Mouse	*Ccl4 (=MIP-1β)*	Mm00443111_m1	Life technologies

Table S2: Histopathological findings

Groups	Presence of inflammation and location	Type of inflammatory infiltrate	Inflammation intensity	Perivascular oedema
Control group (7 mice)	14.3% (1/7) Perivascular and bronchiolitis in 1 case (+ subpleural lymphoid tissue)	Lymphocytes and plasma-cells	- Mild: 14.3% (1/7)<br>- Moderate: 0%<br>- Severe: 0%	Absence
Spore group (7 mice)	87.5% (6/7) Bronchiolitis, alveolitis and perivascular in 6 cases	Lymphocytes, plasma-cells and rare neutrophils/eosinophils	- Mild: 14.3% (1/6)<br>- Moderate: 71.4% (4/6)<br>- Severe: 14.3% (1/6)	100% (7/7)

Table S3. Summary of inflammatory mediators increases in BAL of mice exposed to spores compared to controls, with fold increases and p-values.

**Inflammatory mediators**	**Fold increase**	***p-value***
TNF-α	1.33	0.0087
CXCL-1	2	0.0155
IL-1β	1.5	0.0052
IL-6	5	<0.0001
IL-4	12	0.0006
IL-5	14	0.0012
IL-9	2	0.0261
IL-10	2.33	0.0157
IL-12(p70)	4	0.0004
IL-13	1.77	0.0277
IL-17A	2.55	0.0023
Eotaxin	5	0.0061
G-CSF	4.7	0.0041
IFN-γ	2	0.0495
MCP-1	1.5	0.0064
MIP-1α	6.6	0.0070
MIP-1β	36	0.0006

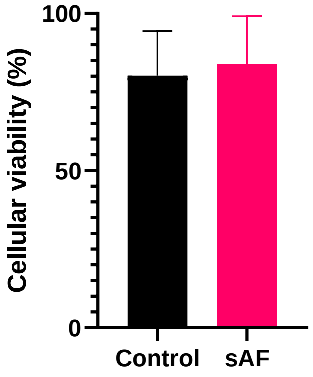

Figure S1: Percentage of cellular viability on co-cultured cells. No statistical difference between control cells and cells challenged with AFsp (p= 0.7326).

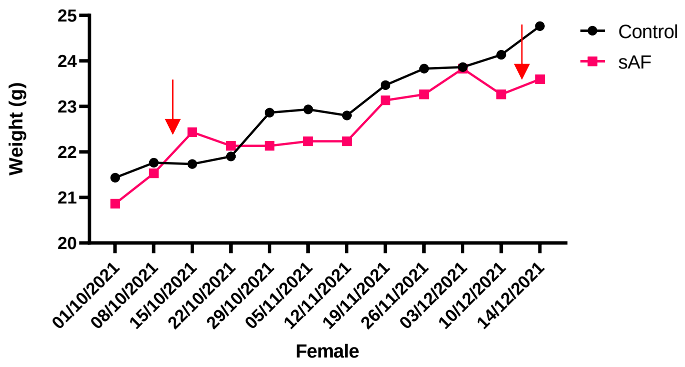

A

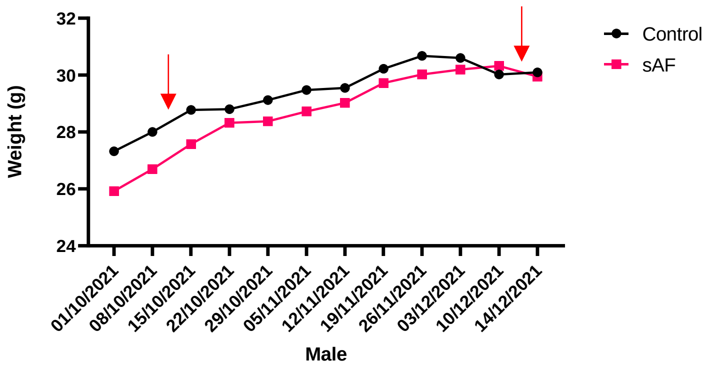

B

Figure S2: Weight of female (A) and male (B) mice after exposure to *Aspergillus fumigatus* spores. No difference between control and challenged mice in body weight gain or lose. Times where instillations were made are indicated with red arrows.

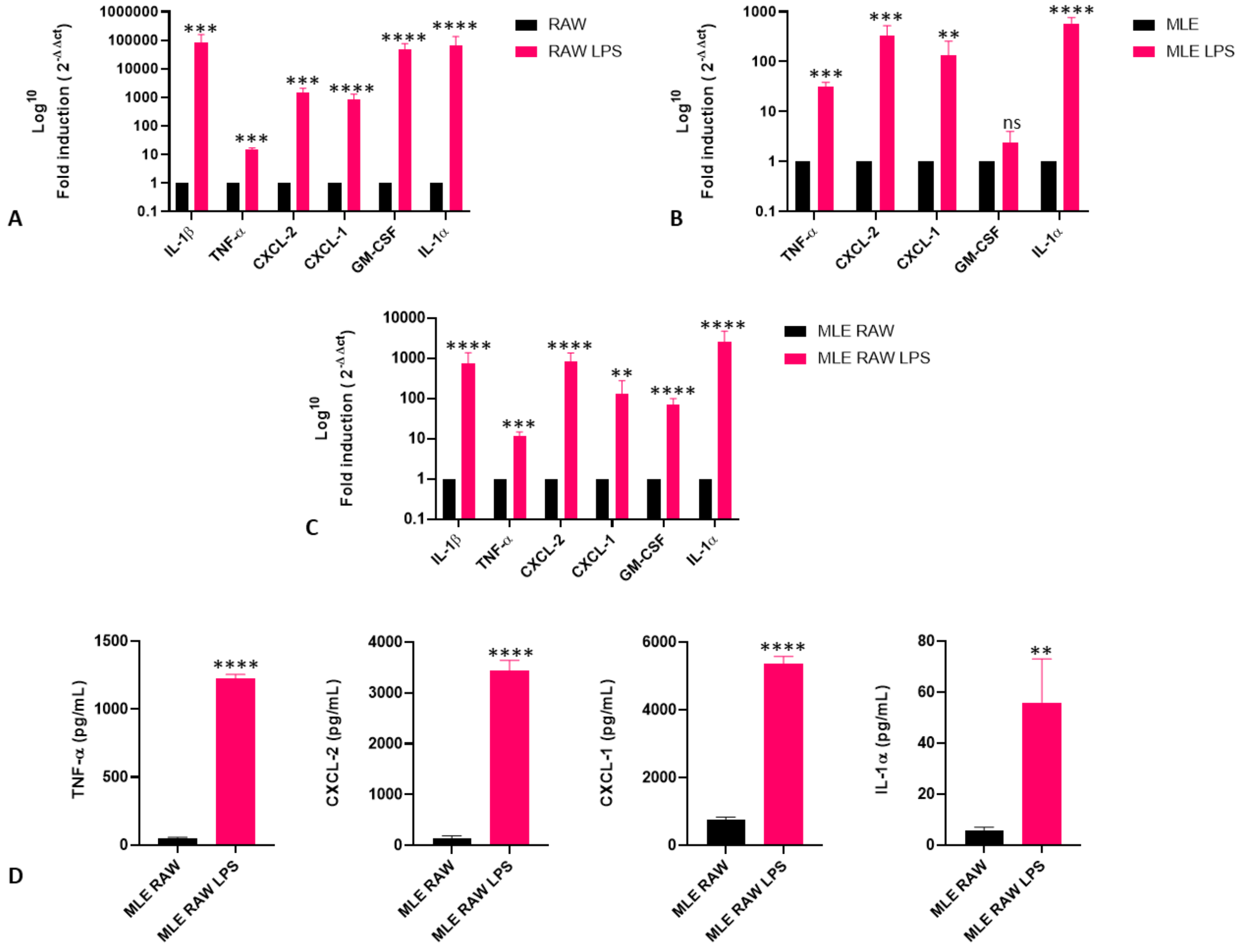

Figure S3: Gene expression and secretion of defined inflammatory mediators after a 4-hour exposure of RAW 264.7, MLE-15 and co-cultured MLE RAW cells to LPS at 1µg/mL (A) Quantification of the gene expression of inflammatory markers in RAW 264.7 cells by RT-qPCR. (B) Quantification of the gene expression of inflammatory markers in MLE-15 cells by RT-qPCR. (C) Quantification of the gene expression of inflammatory markers in co-cultured MLE-15/RAW 264.7 cells by RT-qPCR. (D) Cytokine secretion in the supernatants. Data are represented by mean with SD. (**** $p<0.0001$, ***$p<0.001$, **$p<0.01$ *versus* untreated cells, *n=3 for monocultures and n=4 for co-culture*)
